# Efficacy of Combined 595‐nm Pulsed Dye Laser and Intralesional Corticosteroids Versus Intralesional Corticosteroids Alone for Treating Postmastectomy Hypertrophic Scars and Keloids in Transgender Men: A Randomized Controlled Trial

**DOI:** 10.1111/jocd.70029

**Published:** 2025-03-04

**Authors:** Suthinee Rutnin, Nawara Sakpuwadol, Tanat Yongpisarn, Cherrin Pomsoong, Amornrut Namasondhi, Teerapong Rattananukrom, Kunlawat Thadanipon

**Affiliations:** ^1^ Division of Dermatology, Faculty of Medicine Ramathibodi Hospital Mahidol University Bangkok Thailand; ^2^ Department of Clinical Epidemiology and Biostatistics, Faculty of Medicine Ramathibodi Hospital Mahidol University Bangkok Thailand

**Keywords:** 595‐nm pulsed dye laser, female‐to‐male transgender person, hypertrophic scars, keloids, post‐mastectomy, transmen

## Abstract

**Background:**

Top surgery masculinizes the chest appearance for transgender men (TM); however, some individuals may experience hypertrophic scars (HTS) or keloids following the surgery.

**Objectives:**

This study aimed to evaluate the efficacy of combined 595‐nm pulsed dye laser (PDL) and intralesional triamcinolone acetonide injection (IL TAC) compared to IL TAC monotherapy for treating HTS and keloids.

**Methods:**

Twenty‐five TM with 35 pairs of bilateral symmetric postmastectomy HTS or keloids were randomly allocated to receive the combined PDL and IL TAC on the scar(s) on one side of the chest and IL TAC monotherapy on the contralateral scar(s) in four monthly treatment sessions. Clinical improvement was evaluated using the Vancouver Scar Scale (VSS). Melanin index, hemoglobin index, and scar roughness were determined before each treatment session and at 1, 3, and 6 months after the last treatment. Participant‐rated satisfaction and adverse events were documented.

**Results:**

After two treatment sessions, scars treated with combined PDL and IL TAC demonstrated significantly greater improvements in the VSS (*p* = 0.012) and melanin index (*p* = 0.004) compared to those treated with IL TAC alone. The superior outcomes of the combined therapy persisted for 3 and 6 months after the end of treatment sessions for the VSS (*p* = 0.001) and melanin index (*p* = 0.048), respectively. Participants reported higher satisfaction for combined PDL and IL TAC than IL TAC monotherapy (*p* = 0.005). No serious or permanent adverse event was reported.

**Conclusion:**

The addition of 595‐nm PDL to IL TAC may provide more favorable outcomes for treating postmastectomy HTS and keloids among TM.

## 1 Introduction

Transgender men (TM) are individuals who have a gender identity as male but were assigned female at birth [1]. Several reports have revealed that a great number of TM have experienced chest dysphoria (i.e., feeling distressed from unwanted breast development), which affected their emotional and psychological well‐being [2, 3]. Top surgery is a crucial gender affirmation surgery (GAS) for TM, aiming to masculinize breast appearance, alleviate chest dysphoria, and enhance patients' quality of life. The process involves bilateral subcutaneous mastectomy, nipple‐areola complex reduction, and/or chest contouring surgery [4, 5]. While this procedure is pivotal in aligning one's physical characteristics with their gender identity, the occurrence of post‐surgical hypertrophic scars (HTS) and keloids remains a substantial concern. Particularly, the scars are located on the anterior chest, which is a region prone to stretch and at increased risk for pathological scar formation [6].

HTS and keloids are the results of an abnormal wound healing process [7]. Clinically, HTS is confined to the primary wound margin and usually regresses within a year. Keloid, on the other hand, grows beyond lateral wound boundaries and remains years after initial cutaneous trauma with a high recurrence rate [8, 9]. From our previous study, HTS and keloids were observed in 24% and 4% of TM underwent GAS, respectively [10]. These complications negatively impact the aesthetic outcomes, leading to a decrease in patient satisfaction and quality of life [11]. Several therapies have been introduced, including topical silicone gel sheets, pressure therapy, intralesional injectables such as corticosteroids and 5‐fluorouracil, surgical excision, and laser devices. However, HTS and keloids still pose a significant challenge for physicians since no therapy has proven fully effective and suitable for all lesions [12].

Intralesional corticosteroid injection has been the main treatment for HTS and keloids, with response rates ranging from 50% to 100% and recurrence rates of approximately 9%–50% [12]. Despite its potential, steroids are associated with adverse effects, both local (e.g., skin atrophy, telangiectasia, and dyspigmentation) and systemic effects (e.g., Cushing's syndrome and adrenal insufficiency) [13]. Pulsed dye laser (PDL), a non‐ablative laser device, has emerged as an effective energy‐based device for scar management. The treatment modality works by destroying microvasculature, modulating neocollagenesis, as well as decreasing fibroblast proliferation and growth factors' functions [14]. Studies have found that laser therapy alone has a high recurrence rate at 6–24 months [13]; however, the role of PDL as an adjunctive treatment modality to intralesional medication is promising and remains to be explored [15, 16]. To date, there have been no randomized controlled trials that have evaluated the effectiveness and safety of the adjuvant PDL treatment in addition to IL TAC, with IL TAC serving as a control group, in the treatment of HTS or keloids. Hereby, we aimed to evaluate the efficacy and safety of a combined 595‐nm PDL and intralesional triamcinolone acetonide injection (IL TAC) in comparison to IL TAC monotherapy for treating postmastectomy HTS and keloids in TM.

## 2 Materials and Methods

### 2.1 Study Design

This study was a prospective, evaluator‐blinded, intraindividual split‐sided, randomized controlled trial conducted at a university‐based hospital from November 2021 to December 2022. The protocol conformed to the guidelines of the Declaration of Helsinki. Written informed consent was obtained from all participants prior to enrollment.

### 2.2 Participants

Twenty‐five TM with 35 pairs of symmetric postmastectomy HTS or keloids at the inframammary areas or nipples were enrolled in the study. All participants had received testosterone as their gender‐affirming hormone therapy. Exclusion criteria included a history of previous topical agents, injectables, or laser treatments within 3 months before the study, active skin infection at the area planned for treatment, immunocompromised status, pregnancy, and lactation.

### 2.3 Treatment

**FIGURE 1:**
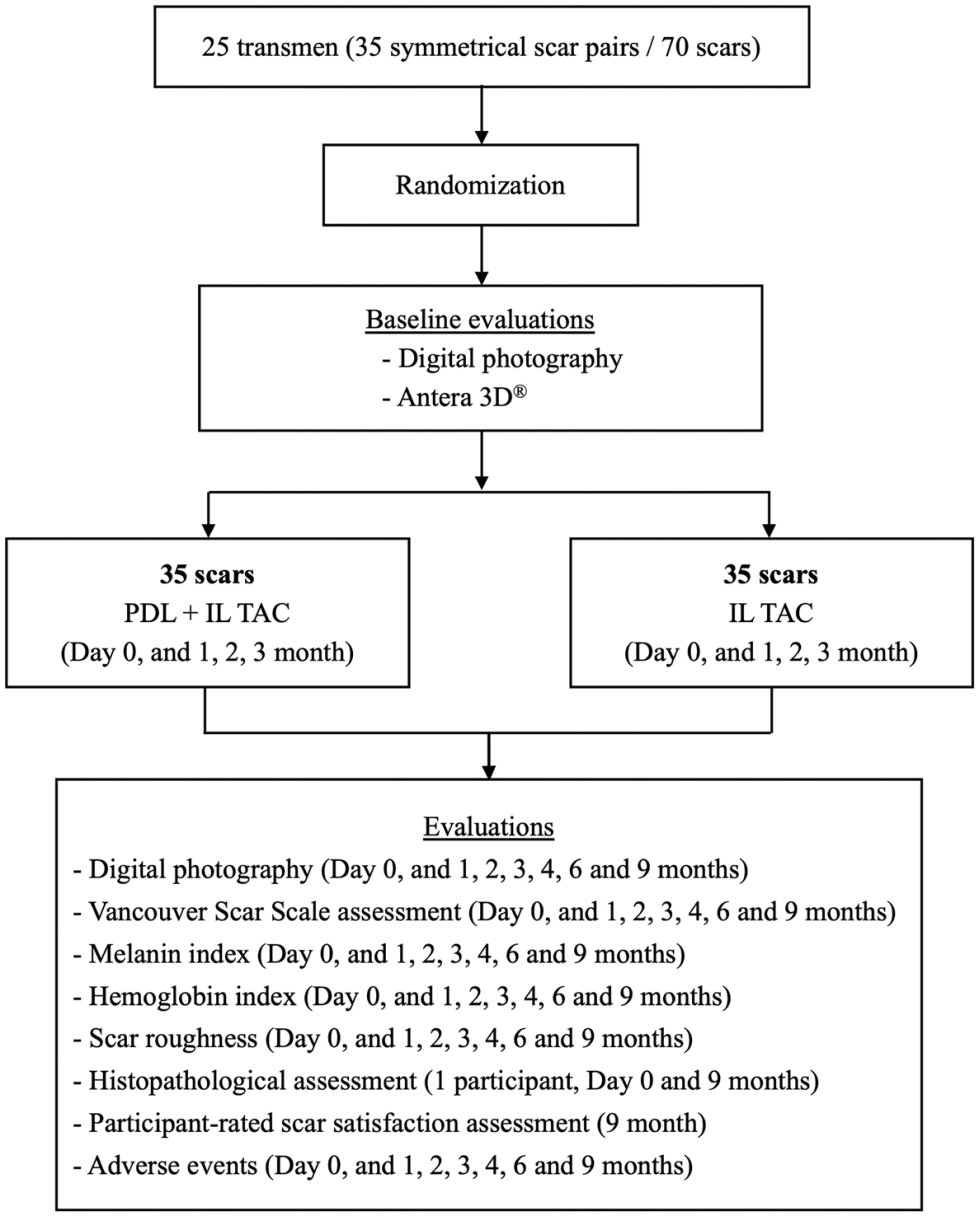
Study flow chart.

A computer‐generated, simple random sequence of the side to receive combined treatment (i.e., PDL and IL TAC) was concealed in sequentially numbered, opaque, sealed envelopes. According to this random sequence, each participant's scar(s) on one side of the chest was/were assigned by an investigator (NS) to receive TAC (Triamcinolone acetonide, TAC; Kenacort, Bristol Myers Squibb, Midtown Manhattan, NYC) combined with 595‐nm PDL (Vbeam Prima, Candela, Marlborough, MA), whereas the contralateral scar(s) was/were treated with IL TAC monotherapy and labeled as control. PDL was performed with a 7‐mm spot size, 5–7 J/cm^2^ fluence, 0.45 ms pulse width, single pass, and 10% overlap. The clinical endpoint of laser therapy was mild to moderate erythema. Following laser irradiation, TAC at 10 mg/mL concentration was injected into the scar using a 30‐gauge needle. The amount of TAC used ranged from 0.3 to 1 mL depending on individual scar sizes, until a slight blanching endpoint was achieved. All patients attended four treatment sessions at 1‐month intervals. Ice pack compression was applied to the area treated with laser to minimize post‐treatment discomfort and burning sensation. The follow‐up time points were 1, 3, and 6 months after the end of the treatment sessions. The study flow is illustrated in Figure 1.

### 2.4 Outcome Evaluation

The primary outcome was the clinical improvement of scars, using the Vancouver Scar Scale (VSS). The VSS is an assessment tool utilized for rating scar appearance based on pigmentation, vascularity, pliability, and height (Table S1). One board‐certified dermatologist, who was blinded to treatment allocation, evaluated the VSS on‐site at every visit.

Secondary outcomes were the improvements in melanin index, hemoglobin index, and scar roughness, measured with a three‐dimensional imaging device (Antera 3D, Miravex Limited, Dublin, Ireland). At the end of the study, participants rated their satisfaction with their scar appearance using a 10‐cm visual analog scale that ranged from not satisfied to extremely satisfied. Adverse events were also documented at every visit.

For histopathological analysis, a punch biopsy was performed on the keloid treated with combined therapy from one volunteer participant at baseline and 6 months after the last treatment. Obtained tissues were stained with hematoxylin and eosin, Verhoeff‐Van Gieson (VVG), and Alcian blue pH 2.5 to assess the changes in collagen bundles, elastic fibers, and mucin deposition, respectively. A blinded dermatopathologist evaluated and reported the histology.

### 2.5 Statistical Analysis

Categorial data were expressed as numbers and percentages, and continuous data were presented as mean and standard deviation (SD) or median and interquartile range (IQR). Mixed‐effects linear regression was used to analyze the VSS, melanin index, hemoglobin index, and scar roughness between the two treatment groups. Participant‐rated satisfaction was compared using a paired *t*‐test. Two‐sided *p* values < 0.05 were considered statistically significant. All data were analyzed using Stata/SE version 16 (StataCorp LLC. College Station, TX).

## 3 Results

### 3.1 Characteristics of Participants

Twenty‐five TM with 35 symmetric pairs of postmastectomy HTS or keloids (15 nipple HTS, 12 inframammary HTS, 2 nipple keloid, and 6 inframammary keloid pairs) were enrolled and completed the study. The mean ± SD age was 32.8 ± 6.91 years. Seven participants had Fitzpatrick skin type III (28%) and 18 had skin type IV (72%). Baseline scar characteristics as measured with VSS and Antera 3D were not significantly different between scars that received combined treatment and controls. The median scar duration was 20 (IQR 11, 57) months. Table 1 displays the characteristics of the patients.

**TABLE 1:** Characteristics of participants.

Characteristic	Value
Age, mean ± SD, years	32.8 ± 6.91
Number of scar's pair per patient, *n* ^a^ (%)	
One pair	15 (60%)
Two pairs	10 (40%)
Fitzpatrick skin type, *n* ^a^ (%)	
III	7 (28%)
IV	18 (72%)
Testosterone replacement therapy, *n* ^a^ (%)	25 (100%)
Scar location and type, *n* ^b^ (%)	
Nipple hypertrophic scars	15 (42.86%)
Inframammary hypertrophic scars	12 (34.29%)
Nipple keloids	2 (5.71%)
Inframammary keloids	6 (17.14%)
Scar duration, median (IQR), months	20 (11, 57)
Previous treatment, *n* ^a^ (%)	
IL TAC	11 (44%)
IL TAC and silicone gel sheet	1 (4%)
Silicone gel sheet	2 (8%)
Topical silicone gel	4 (16%)
Baseline characteristics of scars, mean ± SD VSS	
PDL + IL TAC	7.457 ± 2.29
IL TAC	7.314 ± 2.36
Melanin index	
PDL + IL TAC	0.678 ± 0.08
IL TAC	0.675 ± 0.08
Hemoglobin index	
PDL + IL TAC	1.683 ± 0.33
IL TAC	1.635 ± 0.25
Scar roughness	
PDL + IL TAC	46.256 ± 17.84
IL TAC	48.909 ± 19.28

Abbreviations: IL TAC, intralesional triamcinolone acetonide injection; IQR, interquartile range; PDL, 595‐nm pulsed dye laser; SD, standard deviation; VSS, Vancouver scar scale.

^a^ Indicated the number of participants.

^b^ Indicated the number of pairs of symmetrical mastectomy scars.

### 3.2 Vancouver Scar Scale

**FIGURE 2:**
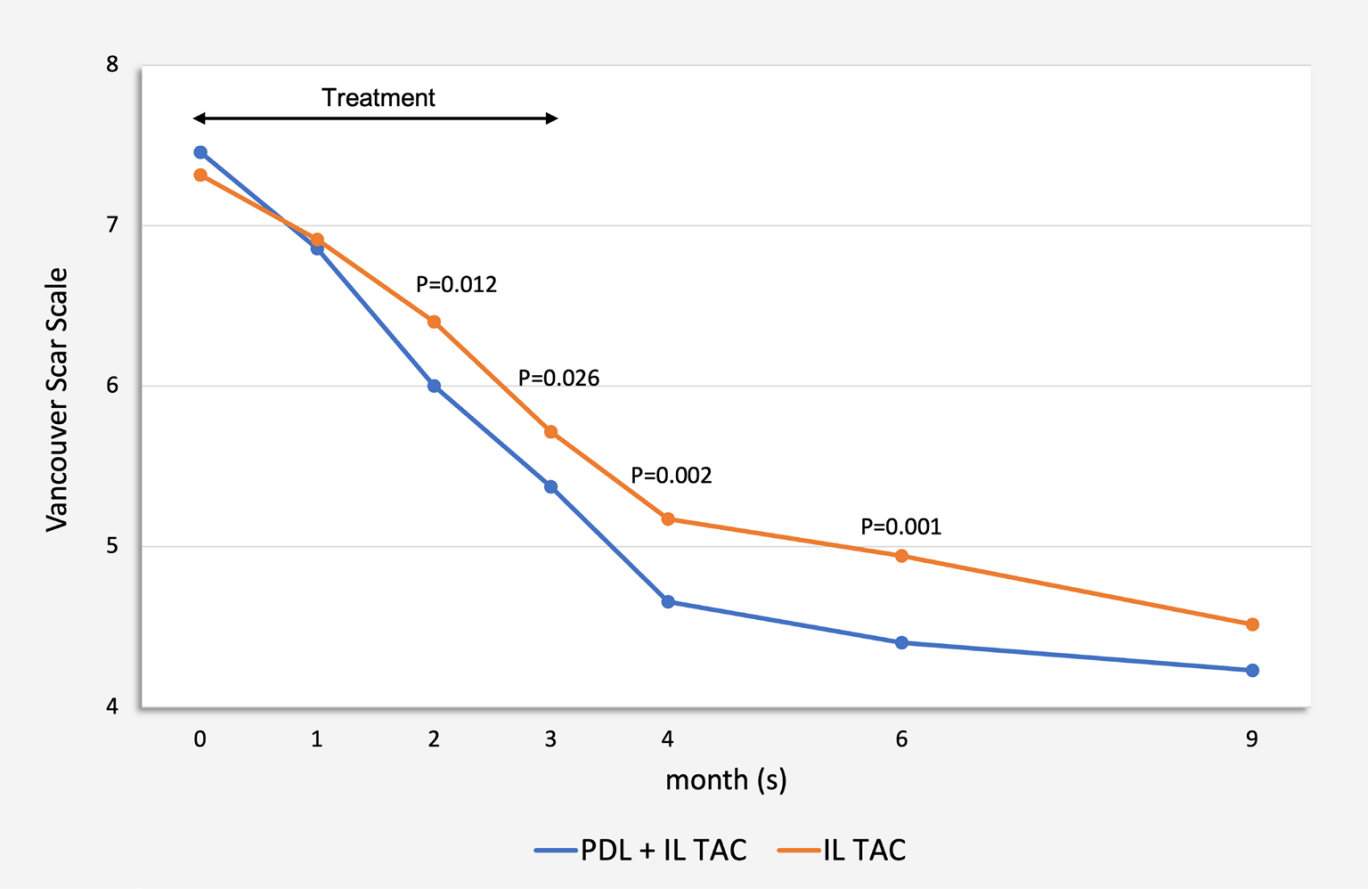
Comparison in mean Vancouver Scar Scale between 595‐nm pulsed dye laser combined with intralesional triamcinolone acetonide‐treated and intralesional triamcinolone acetonide‐treated scars at each visit.

**FIGURE 3:**
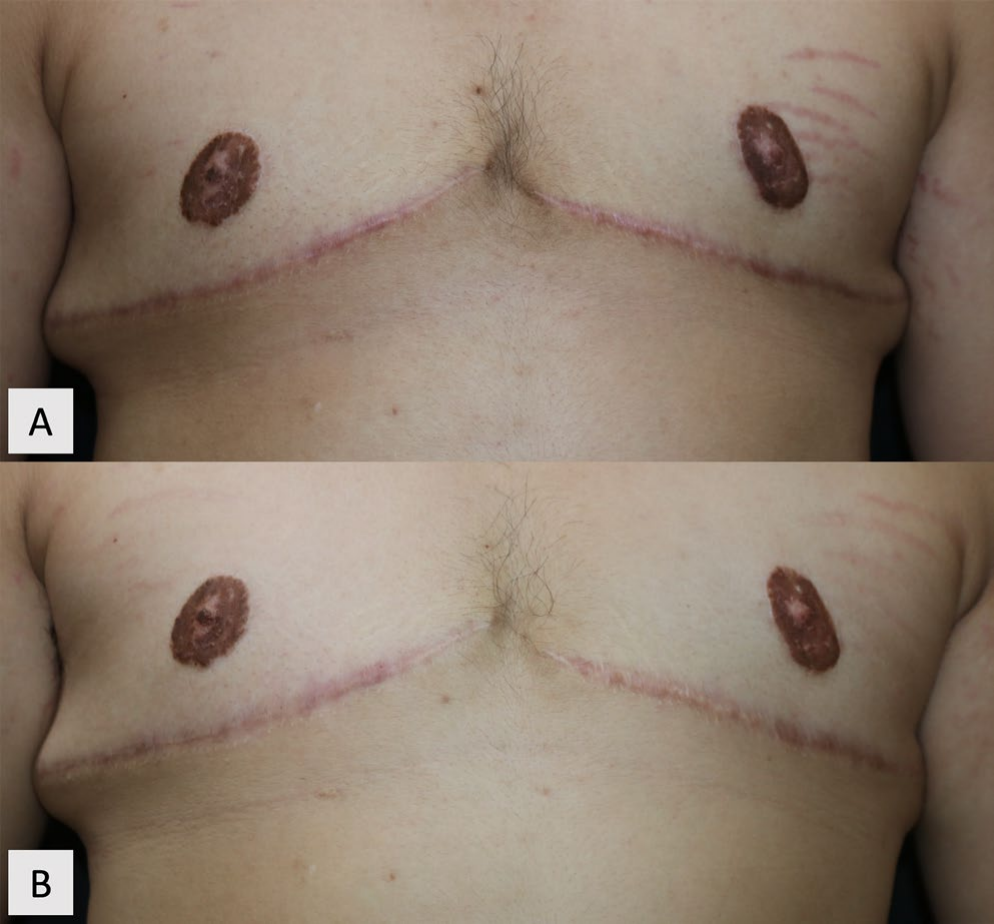
A 24‐year‐old transgender man with symmetric, inframammary, hypertrophic scars aged 10 months at (A) baseline and at (B) 3 months after being treated with four monthly 595‐nm pulsed dye laser combined with intralesional triamcinolone acetonide on the right scar, and four monthly intralesional triamcinolone acetonide on the left scar.

Scars treated with combined PDL and IL TAC showed significantly greater reductions in VSS compared to controls after two treatment sessions (*p* = 0.012) and persisted to 1 and 3 months after the last treatment (*p* = 0.002 and *p* = 0.001 at 1 and 3 months after the final treatment, respectively). The comparison of mean VSS between the two treatment groups is shown in Figure 2; Table S2. Clinical improvement of the mastectomy HTS at baseline and at 3 months after the end of treatment is exemplified in Figure 3.

### 3.3 Melanin Index, Hemoglobin Index, and Scar Roughness

**FIGURE 4:**
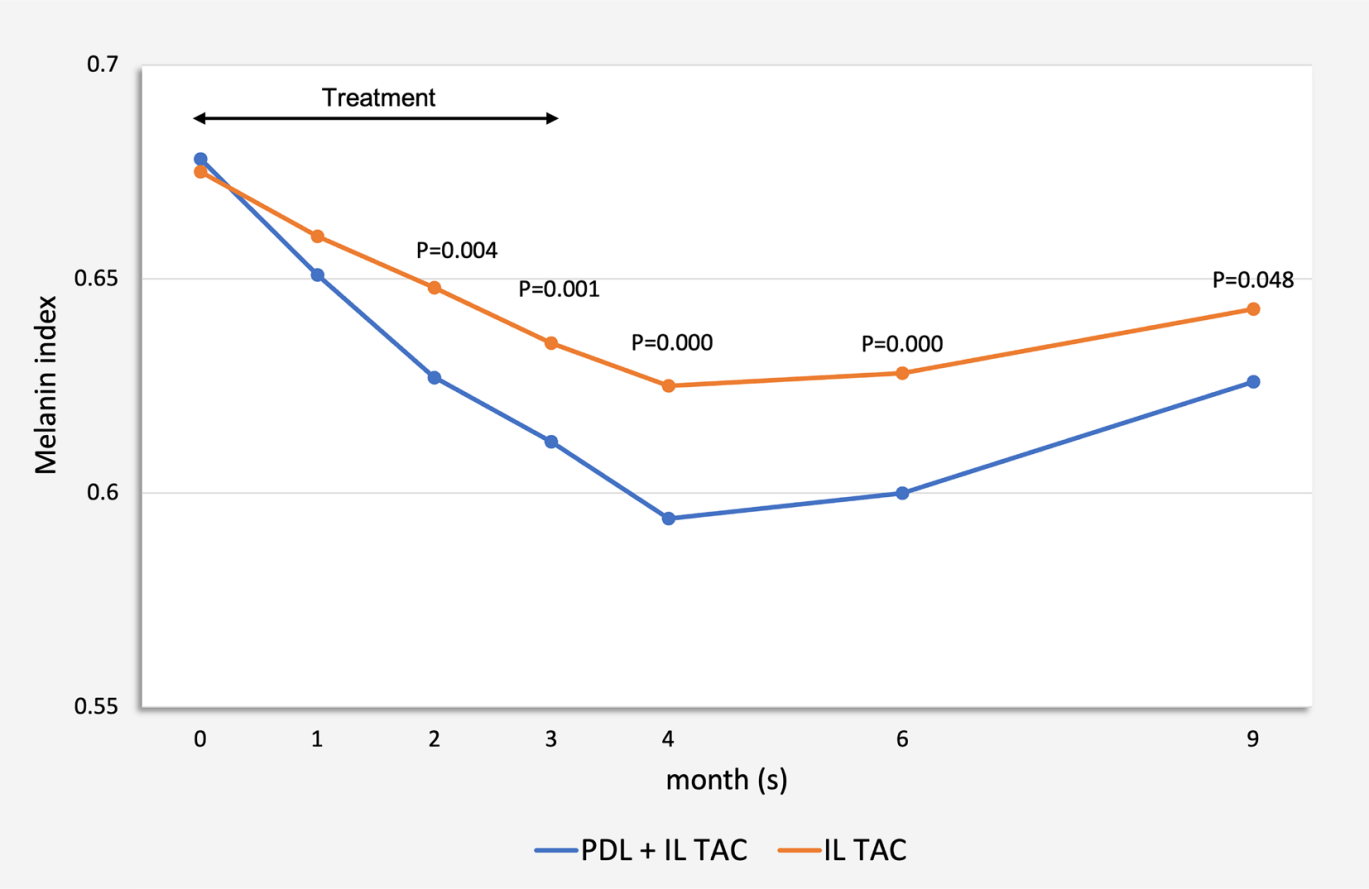
Comparison in mean melanin index between 595‐nm pulsed dye laser combined with intralesional triamcinolone acetonide‐treated and intralesional triamcinolone acetonide‐treated scars at each visit.

After two treatment sessions, combined PDL and IL TAC‐treated scars revealed a statistically significantly lower melanin index than controls (*p* = 0.004). This observation was consistent throughout all follow‐up visits (*p* = 0.001 at after three treatment sessions, and *p* = 0.000, 0.000, 0.048 at 1, 3, and 6 months after the last treatment). Table S3 and Figure 4 show the comparison of the mean melanin index between the combined therapy and monotherapy at each visit. In terms of hemoglobin index and scar roughness, there was no statistically significant difference between the two treatment groups at any visit (Tables S4 and S5).

### 3.4 Participant‐Rated Scar Satisfaction

At the end of the study, participants expressed higher satisfaction with scars that received combined PDL and IL TAC than scars treated with IL TAC alone (*p* = 0.005). The mean ± SD score was 7.448 ± 2.01 for the combined treatment and 6.460 ± 1.80 for the controls.

### 3.5 Adverse Events

No serious or permanent adverse event was reported in our study. Post‐laser purpura was noted in 3.6% of all treatment sessions (5/140), reported by five individuals on the scars treated with the PDL and IL TAC combination. The purpura completely subsided in 7–10 days without dyspigmentation. The occurrence of telangiectasia and skin atrophy was not significantly different between the two groups. Telangiectasia was found in 13 (37.1%) and 12 (34.3%) scars, while skin atrophy in 21 (60%) and 23 (65.7%) scars from the combined therapy and IL TAC monotherapy groups, respectively. No infection was observed during the entire study period.

### 3.6 Histopathological Assessment

**FIGURE 5:**
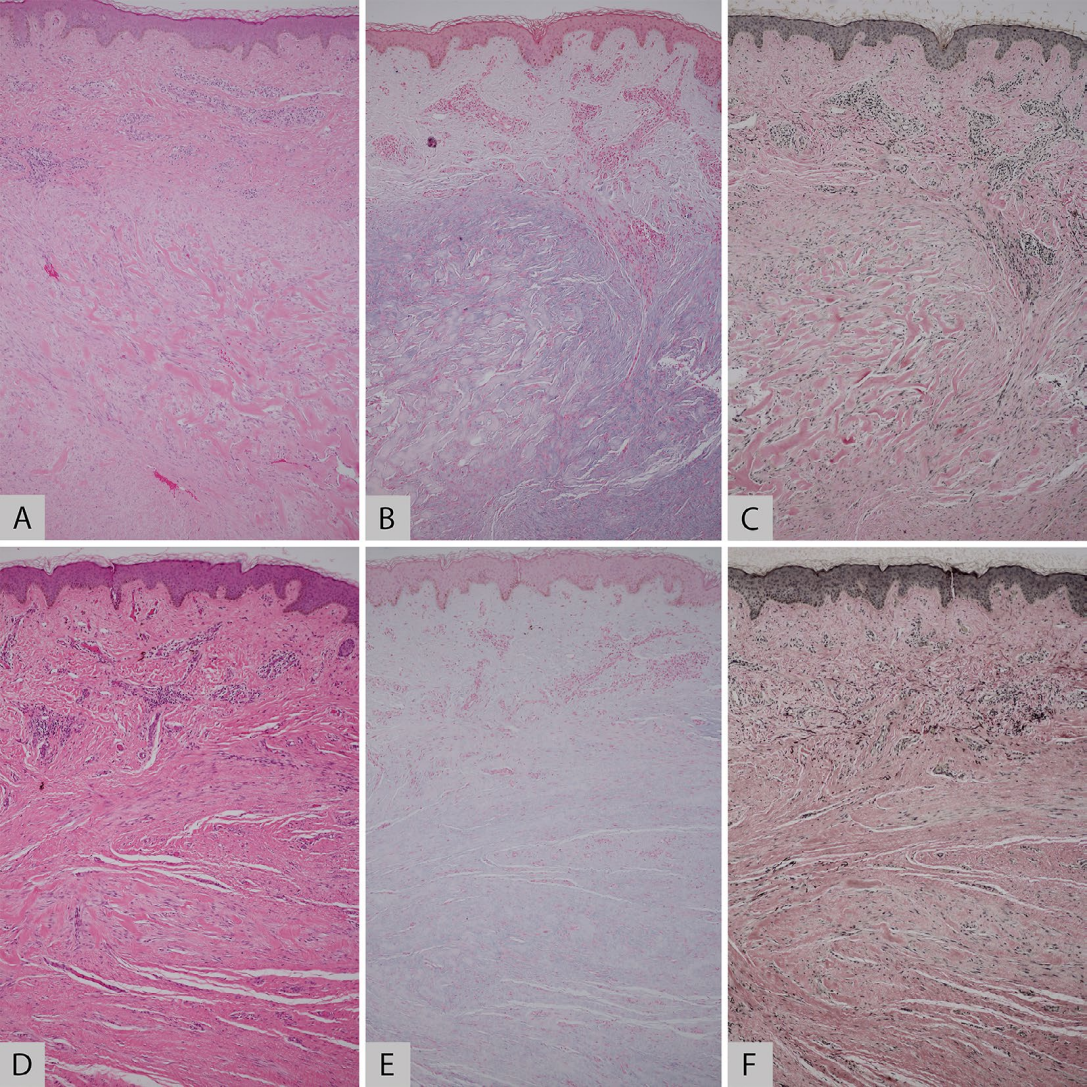
Histopathological assessment obtained from inframammary keloid, at baseline (A–C) and 6 months (D–F) after receiving combined 595‐nm pulsed dye laser and intralesional triamcinolone acetonide on hemotoxylin and eosin (A and D), Verhoeff‐van Gieson (B and E), and Alcian blue pH 2.5 (C and F) staining. (×100 original magnification).

Before receiving combined PDL and IL TAC therapy, the thick and dense keloidal collagens in a haphazard arrangement were observed in the reticular dermis. Six months after four treatment sessions, a reduction in keloidal collagens, and more fine wavy interwoven collagen bundles parallel to the epidermis, representing neocollagenesis, were detected. The VVG stain revealed an increased density of elastic fibers, arranged in a random array, indicating new elastic fiber formation. While the Alcian blue pH 2.5 stain showed a slight increase in mucin deposition (Figure 5).

## 4 Discussion

In the present study, we conducted an evaluator‐blinded, intraindividual split‐sided, randomized, controlled trial to assess the efficacy of combined PDL and IL TAC in comparison to IL TAC monotherapy for treating postmastectomy HTS and keloids in TM. After two treatment sessions, scars receiving combined therapy showed significantly greater VSS improvement than those treated solely with IL TAC (*p* = 0.012), which continued up to 3 months after the final treatment. Moreover, there was a trend toward a significant difference between the two groups at 6 months post‐treatment (*p* = 0.052). In line with the VSS results, better improvement in melanin index from combined therapy was observed after two treatment sessions (*p* = 0.004) and persisted until the end of the study. Our study highlighted the further advantages of integrating PDL with IL TAC in the treatment of HTS and keloids, particularly in terms of the VSS and melanin index.

There have been a few previous reports that explored the utilization of PDL in conjunction with IL steroids. Liu et al. conducted a retrospective study on 29 patients with 39 keloids and found that the group that received both PDL and IL TAC was found to be more efficacious and could reduce the number of IL TAC injection compared with IL TAC alone [17]. Goppold et al. treated two cases of breast reduction HTS with four sessions of 595‐nm PDL and IL TAC at 6‐week intervals, resulting in a significant reduction of scar erythema and height, which remained stable for 2 years without recurrence [18]. Connell et al. observed 10%–95% improvement in scar height and 20%–60% improvement in scar erythema in 10 patients with keloid after performing 2–10 sessions of combined therapy [19]. According to an 18‐week trial by Alster, the use of IL TAC in addition to two sessions of 585‐nm PDL every 6 weeks did not provide any extra advantages relative to PDL alone with regard to clinical improvement or scar pliability in 22 female with bilateral inframammary HTS [20]. However, these existing studies were limited by either their retrospective nature, small sample sizes, or the absence of IL TAC as a control, which yielded inconclusive results in determining the additional benefits of combined PDL and IL TAC versus IL TAC monotherapy.

The precise mechanism through which PDL influences the scars has yet to be elucidated. According to the mechanism of PDL, it selectively targets and destroys microvasculature, thereby inhibiting scar growth by disrupting the nutrient supply to the scars [14]. Some studies propose that PDL may stimulate neocollagenesis, and also inhibit the expression of growth factors that are essential for fibroblast proliferation and extracellular matrix synthesis such as transforming growth factor beta‐1 and connective tissue growth factor, contributing to the improvements of scars [21, 22, 23, 24]. Similar to an earlier report [20], our specimens displayed a decrease in keloidal collagens and an increase in more loosely organized, interwoven collagens and elastic fibers following the combined PDL and IL TAC treatment (Figure 5). In addition, our findings corresponded to previous studies that assessed the histologic improvements of scars following PDL irradiation [25, 26]. This observation aligns with the notion that the scar improvement following PDL treatment may be attributed to the process of collagen remodeling and the formation of new collagen and elastic fibers.

Although 595‐nm PDL was recognized for its primary targeting of hemoglobin, our study revealed a greater impact on the melanin index. This deviation could be due to the fact that this wavelength is also well‐absorbed by melanin [27]. Furthermore, the pulse width employed in the study (i.e., 0.45 ms) was shorter than the epidermal melanin's thermal relaxation time, inevitably subjecting melanin chromophores to the influence of laser energy. Our study was conducted in participants of Asian ethnicity, mainly with skin type IV and greater quantities of epidermal melanin contents, which could therefore absorb light energy competitively against hemoglobin. This finding was consistent with previous studies conducted in the Asian population that reported insignificant differences in scar erythema between PDL‐treated scars and untreated control [28, 29]. We postulate that the combined 595‐nm PDL and IL TAC treatment may exert a more substantial effect on reducing the darkness of scars rather than addressing the redness if performed in darker‐complexioned individuals.

Throughout the study period, the combination of PDL and IL TAC displayed a favorable safety profile. No serious or permanent adverse events were reported. Out of the 140 treatment sessions, only 3.6% (5/140) experienced post‐laser purpura, which completely subsided within 7–10 days without dyspigmentation. Incidences of telangiectasia and skin atrophy were comparable between the combined therapy and control groups, consistent with the recognized side effects of IL steroid injection. The low incidence of adverse events could be attributed to the non‐ablative nature of PDL. Previous studies using ablative and fractional ablative and non‐ablative lasers, such as carbon dioxide laser, fractional carbon dioxide laser, and fractional erbium‐glass laser, reported various adverse events including severe pain, post‐inflammatory hyperpigmentation, hypopigmentation, and infection [30, 31, 32, 33]. Regarding participant‐rated scar satisfaction in our study, patients were more pleased with scars treated with combined PDL and IL TAC. This emphasizes the effectiveness, safety, and tolerability of PDL as a laser device suitable to be used in conjunction with standard treatment for addressing HTS and keloids.

To the best of our knowledge, this study marks the first randomized controlled trial evaluating the efficacy of the combination of 595‐nm PDL and IL TAC compared to IL TAC monotherapy for treating postmastectomy HTS and keloids among TM. There were some limitations in our study. First, a small sample size that lacked sufficient power to perform subgroup analyses for each scar type. Second, all recruited scars were located on the anterior chest, therefore limiting the generalizability of our findings to the different locations. Third, the histological analysis was conducted on only one participant with keloid, which may not be sufficient evidence to reliably support the findings of this study. Lastly, all included TM received testosterone as part of their gender‐affirming therapy, which could potentially influence the wound healing process and impair scar formation [34].

## 5 Conclusion

The addition of 595‐nm PDL to IL TAC may provide more favorable outcomes for treating postmastectomy HTS and keloids among TM.

## Author Contributions

S.R., and K.T. designed the study. N.S., T.Y., C.P., and A.N. carried out the treatments and measurements. S.R., T.R., and K.T. contributed to the evaluation and interpretation of the results. N.S. and T.Y. wrote the manuscript. S.R., and K.T. supervised the project.

## Conflicts of Interest

The authors have stated explicitly that there are no conflicts of interest in connection with this article.

## Study Design

This study was a prospective, evaluator‐blinded, intraindividual split‐sided, randomized controlled trial conducted at a university‐based hospital (Ramathibodi Hospital, Mahidol University, Bangkok, Thailand) from November 2021 to December 2022. The study was approved by the Institutional Review Board for Human Subject Research, Faculty of Medicine Ramathibodi Hospital, Mahidol University (protocol number COA. MURA2021/244) and registered with the Thai Clinical Trials Registry (identification number TCTR20230304002).

## Supporting information


Table S1.

## Data Availability

The data that support the findings of this study are available from the corresponding author upon reasonable request.

## References

[1] E. Coleman , A. E. Radix , W. P. Bouman , et al., “Standards of Care for the Health of Transgender and Gender Diverse People, Version 8,” International Journal of Transgender Health 23, no. S1 (2022): S260. 10.1080/26895269.2022.2100644 PMC9553112 36238954

[2] M. Ascha , D. C. Sasson , R. Sood , et al., “Top Surgery and Chest Dysphoria Among Transmasculine and Nonbinary Adolescents and Young Adults,” JAMA Pediatrics 176 (2022): 1115–1122. 36156703 10.1001/jamapediatrics.2022.3424 PMC9513704

[3] J. Olson‐Kennedy , J. Warus , V. Okonta , M. Belzer , and L. F. Clark , “Chest Reconstruction and Chest Dysphoria in Transmasculine Minors and Young Adults: Comparisons of Nonsurgical and Postsurgical Cohorts,” JAMA Pediatrics 172 (2018): 431–436. 29507933 10.1001/jamapediatrics.2017.5440 PMC5875384

[4] C. A. Agarwal , M. F. Scheefer , L. N. Wright , N. K. Walzer , and A. Rivera , “Quality of Life Improvement After Chest Wall Masculinization in Female‐to‐Male Transgender Patients: A Prospective Study Using the BREAST‐Q and Body Uneasiness Test,” Journal of Plastic, Reconstructive & Aesthetic Surgery 71 (2018): 651–657. 10.1016/j.bjps.2018.01.003 29422399

[5] R. Weigert , E. Frison , Q. Sessiecq , K. Al Mutairi , and V. Casoli , “Patient Satisfaction With Breasts and Psychosocial, Sexual, and Physical Well‐Being After Breast Augmentation in Male‐to‐Female Transsexuals,” Plastic and Reconstructive Surgery 132 (2013): 1421–1429. 24281571 10.1097/01.prs.0000434415.70711.49

[6] R. Ogawa and S. Akaishi , “Endothelial Dysfunction May Play a Key Role in Keloid and Hypertrophic Scar Pathogenesis–Keloids and Hypertrophic Scars May Be Vascular Disorders,” Medical Hypotheses 96 (2016): 51–60. 27959277 10.1016/j.mehy.2016.09.024

[7] B. Berman , A. Maderal , and B. Raphael , “Keloids and Hypertrophic Scars: Pathophysiology, Classification, and Treatment,” Dermatologic Surgery 43, no. 1 (2017): S3–S18. 27347634 10.1097/DSS.0000000000000819

[8] F. M. Ghazawi , R. Zargham , M. S. Gilardino , D. Sasseville , and F. Jafarian , “Insights Into the Pathophysiology of Hypertrophic Scars and Keloids: How Do They Differ?,” Advances in Skin & Wound Care 31 (2018): 582–595. 29240586 10.1097/01.ASW.0000527576.27489.0f

[9] S. F. Ekstein , S. P. Wyles , S. L. Moran , and A. Meves , “Keloids: A Review of Therapeutic Management,” International Journal of Dermatology 60 (2021): 661–671. 32905614 10.1111/ijd.15159 PMC7940466

[10] S. Rutnin , P. Suchonwanit , C. Kositkuljorn , et al., “Characterizing Dermatological Conditions in the Transgender Population: A Cross‐Sectional Study,” Transgend Health 8 (2023): 89–99. 36824384 10.1089/trgh.2021.0105 PMC9942180

[11] J. M. Amici , C. Taieb , C. Le Floc'h , A. Demessant , S. Seité , and O. Cogrel , “The Impact of Visible Scars on Well‐Being and Quality of Life: An International Epidemiological Survey in Adults,” Journal of the European Academy of Dermatology and Venereology 37, no. Suppl 3 (2023): 3–6. 10.1111/jdv.18856 36635614

[12] F. S. Frech , L. Hernandez , R. Urbonas , G. A. Zaken , I. Dreyfuss , and K. Nouri , “Hypertrophic Scars and Keloids: Advances in Treatment and Review of Established Therapies,” American Journal of Clinical Dermatology 24 (2023): 225–245. 36662366 10.1007/s40257-022-00744-6

[13] M. Morelli Coppola , R. Salzillo , F. Segreto , and P. Persichetti , “Triamcinolone Acetonide Intralesional Injection for the Treatment of Keloid Scars: Patient Selection and Perspectives,” Clinical, Cosmetic and Investigational Dermatology 11 (2018): 387–396. 30087573 10.2147/CCID.S133672 PMC6063260

[14] S. R. Reiken , S. F. Wolfort , F. Berthiaume , C. Compton , R. G. Tompkins , and M. L. Yarmush , “Control of Hypertrophic Scar Growth Using Selective Photothermolysis,” Lasers in Surgery and Medicine 21 (1997): 7–12. 9228634 10.1002/(sici)1096-9101(1997)21:1<7::aid-lsm2>3.0.co;2-u

[15] A. Asilian , A. Darougheh , and F. Shariati , “New Combination of Triamcinolone, 5‐Fluorouracil, and Pulsed‐Dye Laser for Treatment of Keloid and Hypertrophic Scars,” Dermatologic Surgery 32 (2006): 907–915. 16875473 10.1111/j.1524-4725.2006.32195.x

[16] Z. N. Liu , Y. M. Zhou , R. X. Liu , et al., “Clinical Effects of Pulsed Dye Laser Dynamically Combined With Triamcinolone Acetonide in the Treatment of Keloids,” Zhonghua Shao Shang Yu Chuang Mian Xiu Fu Za Zhi 38 (2022): 822–829. 36177586 10.3760/cma.j.cn501225-20220620-00249 PMC11704680

[17] Z. Liu , J. Zhang , and X. Guo , “Clinical Effects of Pulsed Dye Laser Dynamically Combined With Triamcinolone Acetonide in the Treatment of Postoperativr Recuurence Keloids,” Indian Journal of Dermatology 68 (2023): 486. 10.4103/ijd.ijd_883_22 PMC10564205 37822374

[18] A. Goppold , K. M. Kaune , T. Buhl , M. P. Schön , and M. Zutt , “595nm Pulsed Dye Laser Combined With Intralesional Corticosteroids in Hypertrophic Symptomatic Scars Following Breast Reduction Surgery,” European Journal of Dermatology 21 (2011): 262–263. 21382779 10.1684/ejd.2010.1230

[19] P. G. Connell and C. C. Harland , “Treatment of Keloid Scars With Pulsed Dye Laser and Intralesional Steroid,” Journal of Cutaneous Laser Therapy 2 (2000): 147–150. 11360332 10.1080/14628830050516407

[20] T. Alster , “Laser Scar Revision: Comparison Study of 585‐nm Pulsed Dye Laser With and Without Intralesional Corticosteroids,” Dermatologic Surgery 29 (2003): 25–29. 12534508 10.1046/j.1524-4725.2003.29024.x

[21] R. Zhu , B. Yue , Q. Yang , et al., “The Effect of 595 Nm Pulsed Dye Laser on Connective Tissue Growth Factor (CTGF) Expression in Cultured Keloid Fibroblasts,” Lasers in Surgery and Medicine 47 (2015): 203–209. 25727552 10.1002/lsm.22334

[22] Q. Yang , Y. Ma , R. Zhu , et al., “The Effect of Flashlamp Pulsed Dye Laser on the Expression of Connective Tissue Growth Factor in Keloids,” Lasers in Surgery and Medicine 44 (2012): 377–383. 22539077 10.1002/lsm.22031

[23] Y. R. Kuo , S. F. Jeng , F. S. Wang , et al., “Flashlamp Pulsed Dye Laser (PDL) Suppression of Keloid Proliferation Through Down‐Regulation of TGF‐beta1 Expression and Extracellular Matrix Expression,” Lasers in Surgery and Medicine 34 (2004): 104–108. 15004820 10.1002/lsm.10206

[24] J. Zhang , S. Zhou , Z. Xia , et al., “595‐Nm Pulsed Dye Laser Combined With Fractional CO(2) Laser Reduces Hypertrophic Scar Through Down‐Regulating TGFβ1 and PCNA,” Lasers in Medical Science 36 (2021): 1625–1632. 34117539 10.1007/s10103-020-03240-7

[25] W. Manuskiatti and R. E. Fitzpatrick , “Treatment Response of Keloidal and Hypertrophic Sternotomy Scars: Comparison Among Intralesional Corticosteroid, 5‐Fluorouracil, and 585‐nm Flashlamp‐Pumped Pulsed‐Dye Laser Treatments,” Archives of Dermatology 138 (2002): 1149–1155. 12224975 10.1001/archderm.138.9.1149

[26] H. Liu , Y. Dang , Z. Wang , X. Chai , and Q. Ren , “Laser Induced Collagen Remodeling: A Comparative Study In Vivo on Mouse Model,” Lasers in Surgery and Medicine 40 (2008): 13–19. 18220261 10.1002/lsm.20587

[27] A. K. Tong , O. T. Tan , J. Boll , J. A. Parrish , and G. F. Murphy , “Ultrastructure: Effects of Melanin Pigment on Target Specificity Using a Pulsed Dye Laser (577 nm),” Journal of Investigative Dermatology 88 (1987): 747–752. 3585058 10.1111/1523-1747.ep12470418

[28] W. Manuskiatti , R. E. Fitzpatrick , and M. P. Goldman , “Energy Density and Numbers of Treatment Affect Response of Keloidal and Hypertrophic Sternotomy Scars to the 585‐nm Flashlamp‐Pumped Pulsed‐Dye Laser,” Journal of the American Academy of Dermatology 45 (2001): 557–565. 11568747 10.1067/mjd.2001.116580

[29] W. Manuskiatti , R. Wanitphakdeedecha , and R. E. Fitzpatrick , “Effect of Pulse Width of a 595‐nm Flashlamp‐Pumped Pulsed Dye Laser on the Treatment Response of Keloidal and Hypertrophic Sternotomy Scars,” Dermatologic Surgery 33 (2007): 152–161. 17300600 10.1111/j.1524-4725.2006.33033.x

[30] B. M. El‐Zawahry , R. M. Sobhi , D. A. Bassiouny , and S. A. Tabak , “Ablative CO_2_ Fractional Resurfacing in Treatment of Thermal Burn Scars: An Open‐Label Controlled Clinical and Histopathological Study,” Journal of Cosmetic Dermatology 14 (2015): 324–331. 26260018 10.1111/jocd.12163

[31] Y. Lei , S. F. Li , Y. L. Yu , J. Tan , and M. H. Gold , “Clinical Efficacy of Utilizing Ultrapulse CO(2) Combined With Fractional CO_2_ Laser for the Treatment of Hypertrophic Scars in Asians‐A Prospective Clinical Evaluation,” Journal of Cosmetic Dermatology 16 (2017): 210–216. 29058830 10.1111/jocd.12334

[32] S. Alexander , B. S. Girisha , H. Sripathi , T. M. Noronha , and A. C. Alva , “Efficacy of Fractional CO(2) Laser With Intralesional Steroid Compared With Intralesional Steroid Alone in the Treatment of Keloids and Hypertrophic Scars,” Journal of Cosmetic Dermatology 18 (2019): 1648–1656. 30770627 10.1111/jocd.12887

[33] B. Behera , R. Kumari , D. M. Thappa , and M. Malathi , “Therapeutic Efficacy of Intralesional Steroid With Carbon Dioxide Laser Versus With Cryotherapy in Treatment of Keloids: A Randomized Controlled Trial,” Dermatologic Surgery 42 (2016): 1188–1198. 27661432 10.1097/DSS.0000000000000873

[34] V. Mroueh , E. Reiche , J. Mroueh , et al., “Androgen Therapy Worsens Scar Formation in Masculinizing Mastectomy,” British Journal of Surgery 110 (2023): 1422–1424. 37303282 10.1093/bjs/znad148

