# Supplementary material for: Efficacy of Combined 595‐nm Pulsed Dye Laser and Intralesional Corticosteroids Versus Intralesional Corticosteroids Alone for Treating Postmastectomy Hypertrophic Scars and Keloids in Transgender Men: A Randomized Controlled Trial
Source: J Cosmet Dermatol. 2025 Mar 4;24(3):e70029. doi: 10.1111/jocd.70029 (PMC11877627; doi:10.1111/jocd.70029)
Supplement: Supplementary file 1 — Table S1. [file JOCD-24-e70029-s001.docx]

**Supplementary Table 1** The Vancouver Scar Scale

| **Parameter** | **Descriptor** | **Points** |
| --- | --- | --- |
| **Pigmentation** | Normal | 0 |
|  | Hypopigmentation | 1 |
|  | Hyperpigmentation | 2 |
| **Vascularity** | Normal | 0 |
|  | Pink | 1 |
|  | Red | 2 |
|  | Purple | 3 |
| **Pliability** | Normal | 0 |
|  | Supple – flexible with minimal resistance | 1 |
|  | Yielding – giving way to pressure, offering moderate resistance, but does not behave as a solid scar mass | 2 |
|  | Firm – inflexible not easily moved, resistant to manual pressure | 3 |
|  | Banding – rope-like tissue that blanches with extension of scar | 4 |
|  | Contracture – permanent shortening of scar producing deformity or distortion | 5 |
| **Height** | Normal – flat | 0 |
|  | >0 and <2 mm | 1 |
|  | ≥2 mm and <5 mm | 2 |
|  | ≥5 mm | 3 |
| **Total score** | | /13 |

**Supplementary Table 2** Vancouver Scar Scale of 595-nm pulsed dye laser combined with intralesional triamcinolone acetonide-treated and intralesional triamcinolone acetonide-treated scars assessed by a blinded dermatologist at each visit

| **Visit** | **Month** | **Vancouver scar scale, mean ± SD** | | |
| --- | --- | --- | --- | --- |
|  |  | **PDL + IL TAC** | **IL TAC** | **P-value** |
| 1 (Baseline) | 0 | 7.457 ± 2.29 | 7.314 ± 2.36 | 0.867 |
| 2 | 1 | 6.857 ± 2.43 | 6.914 ± 2.38 | 0.415 |
| 3 | 2 | 6.000 ± 2.20 | 6.400 ± 2.17 | 0.012* |
| 4 | 3 | 5.371 ± 2.19 | 5.714 ± 2.33 | 0.026* |
| 5 | 4 (1 month post-laser) | 4.657 ± 1.81 | 5.171 ± 2.12 | 0.002* |
| 6 | 6 (3 months post-laser) | 4.400 ± 1.76 | 4.942 ± 2.22 | 0.001* |
| 7 | 9 (6 months post-laser) | 4.228 ± 1.81 | 4.514 ± 1.96 | 0.052 |

Abbreviations: IL TAC, intralesional triamcinolone acetonide injection; PDL, 595-nm pulsed-dye laser; SD, standard deviation.

*P-values < 0.05 are considered statistically significant.

**Supplementary Table 3** Melanin index of 595-nm pulsed dye laser combined with intralesional triamcinolone acetonide-treated and intralesional triamcinolone acetonide-treated scars assessed with Antera 3D® at each visit

| **Visit** | **Month** | **Melanin index, mean ± SD** | | |
| --- | --- | --- | --- | --- |
|  |  | **PDL + IL TAC** | **IL TAC** | **P-value** |
| 1 (Baseline) | 0 | 0.678 ± 0.08 | 0.675 ± 0.08 | 0.896 |
| 2 | 1 | 0.651 ± 0.07 | 0.660 ± 0.08 | 0.139 |
| 3 | 2 | 0.627 ± 0.06 | 0.648 ± 0.08 | 0.004* |
| 4 | 3 | 0.612 ± 0.07 | 0.635 ± 0.08 | 0.001* |
| 5 | 4 (1 month post-laser) | 0.594 ± 0.08 | 0.625 ± 0.08 | 0.000* |
| 6 | 6 (3 months post-laser) | 0.600 ± 0.06 | 0.628 ± 0.08 | 0.000* |
| 7 | 9 (6 months post-laser) | 0.626 ± 0.07 | 0.643 ± 0.08 | 0.048* |

Abbreviations: IL TAC, intralesional triamcinolone acetonide injection; PDL, 595-nm pulsed-dye laser; SD, standard deviation.

*P-values < 0.05 are considered statistically significant.

**Supplementary Table 4** Hemoglobin index of 595-nm pulsed dye laser combined with intralesional triamcinolone acetonide-treated and intralesional triamcinolone acetonide-treated scars assessed with Antera 3D® at each visit

| **Visit** | **Month** | **Hemoglobin index, mean ± SD** | | |
| --- | --- | --- | --- | --- |
|  |  | **PDL + IL TAC** | **IL TAC** | **P-value** |
| 1 (Baseline) | 0 | 1.683 ± 0.33 | 1.635 ± 0.25 | 0.696 |
| 2 | 1 | 1.674 ± 0.38 | 1.623 ± 0.31 | 0.419 |
| 3 | 2 | 1.648 ± 0.34 | 1.622 ± 0.30 | 0.723 |
| 4 | 3 | 1.649 ± 0.39 | 1.611 ± 0.34 | 0.825 |
| 5 | 4 (1 month post-laser) | 1.622 ± 0.38 | 1.620 ± 0.31 | 0.244 |
| 6 | 6 (3 months post-laser) | 1.587 ± 0.35 | 1.596 ± 0.33 | 0.141 |
| 7 | 9 (6 months post-laser) | 1.587 ± 0.36 | 1.542 ± 0.31 | 0.632 |

Abbreviations: IL TAC, intralesional triamcinolone acetonide injection; PDL, 595-nm pulsed-dye laser; SD, standard deviation.

*P-values < 0.05 are considered statistically significant.

**Supplementary Table 5** Scar roughness of 595-nm pulsed dye laser combined with intralesional triamcinolone acetonide-treated and intralesional triamcinolone acetonide-treated scars assessed with Antera 3D® at each visit

| **Visit** | **Month** | **Scar roughness, mean ± SD** | | |
| --- | --- | --- | --- | --- |
|  |  | **PDL + IL TAC** | **IL TAC** | **P-value** |
| 1 (Baseline) | 0 | 46.256 ± 17.84 | 48.909 ± 19.28 | 0.691 |
| 2 | 1 | 41.482 ± 16.21 | 43.277 ± 17.02 | 0.905 |
| 3 | 2 | 38.303 ± 12.53 | 38.891 ± 15.34 | 0.556 |
| 4 | 3 | 37.410 ± 13.58 | 39.615 ± 14.61 | 0.677 |
| 5 | 4 (1 month post-laser) | 35.068 ± 13.53 | 38.069 ± 14.89 | 0.455 |
| 6 | 6 (3 months post-laser) | 37.234 ± 14.10 | 38.078 ± 13.28 | 0.809 |
| 7 | 9 (6 months post-laser) | 39.739 ± 13.89 | 39.333 ± 11.55 | 0.703 |

Abbreviations: IL TAC, intralesional triamcinolone acetonide injection; PDL, 595-nm pulsed-dye laser; SD, standard deviation.

*P-values < 0.05 are considered statistically significant.
